# Unveiling Drug-Drug Interactions in Dental Patients: A Retrospective Real-World Study

**DOI:** 10.3390/dj13060255

**Published:** 2025-06-09

**Authors:** Daiana Colibășanu, Sebastian Mihai Ardelean, Florina-Diana Goldiș, Maria-Medana Drăgoi, Sabina-Oana Vasii, Tamara Maksimović, Șerban Colibășanu, Codruța Șoica, Lucreția Udrescu

**Affiliations:** 1 Center for Drug Data Analysis, Cheminformatics, and the Internet of Medical Things, “Victor Babeș” University of Medicine and Pharmacy Timișoara, 300041 Timișoara, Romania; daiana.handa@umft.ro (D.C.); diana.goldis@umft.ro (F.-D.G.); medana.tuica@umft.ro (M.-M.D.); sabina.vasii@umft.ro (S.-O.V.); 2 Departament II–Pharmaceutical Chemistry, “Victor Babeş” University of Medicine and Pharmacy Timisoara, 300041 Timișoara, Romania; 3 Department of Computer and Information Technology, University Politehnica of Timișoara, 300223 Timișoara, Romania; sebastian.ardelean@cs.upt.ro; 4 Departament II–Pharmacology-Pharmacotherapy, “Victor Babeş” University of Medicine and Pharmacy Timisoara, 300041 Timișoara, Romania; tamara.maksimovic@umft.ro (T.M.); codrutasoica@umft.ro (C.Ș.); 5 Research Center for Experimental Pharmacology and Drug Design (X-Pharm Design), “Victor Babeş” University of Medicine and Pharmacy Timisoara, 300041 Timișoara, Romania; 6 Dental Point Clinic, 300724 Timișoara, Romania; colibasanus@gmail.com; 7 Department I–Drug Analysis, “Victor Babeş” University of Medicine and Pharmacy Timişoara, 300041 Timişoara, Romania

**Keywords:** drug-drug interactions, dental pharmacotherapy, real-world data, patient safety, age-related drug interactions

## Abstract

**Background**: Drug-drug interactions (DDIs) are a growing safety concern in dental care, particularly among patients with comorbidities and polypharmacy. However, real-world data (RWD) on the prevalence and severity of DDIs in dental settings remain scarce. **Objectives**: This study aimed to assess the frequency, severity, and clinical relevance of DDIs in dental patients and to identify age- and comorbidity-related risk patterns. **Methods**: This retrospective study analyzed a cohort of 105 dental patients, considering demographics, preexisting diseases, dental procedures, and prescribed medications. We examined drug-drug interactions (DDIs) employing the DrugBank Drug Interaction Checker, which yields DDI severity into major, moderate, or minor. **Results**: 45.7% of patients had preexisting diseases, with cardiovascular diseases most prevalent (19.0%). Higher prevalent dental diagnoses and procedures included apical lesions (47.6%) and tooth extractions (53.3%), suggesting frequent pharmacotherapy exposure. We identified 542 DDIs out of 1332 drug pairs and found 2.3% major, 25.0% moderate, and 13.4% minor, with 59.3% showing no interactions. Key high-risk DDIs included epinephrine with beta-blockers. Fifteen patients aged 31–60 years experienced the most major DDIs of 61.3%, patients ≥ 61 years faced 38.7%, and the 0–30 group had none, highlighting age-specific risks. The higher DDIs burden in the 31–60 age group may reflect better knowledge of the drugs they used and accurate reporting of them. **Conclusions**: Our retrospective study addresses the paucity of dental DDIs real-world data (RWD) studies, pleading for improved drug reconciliation, systematic screening, and age- and comorbidity-tailored strategies to enhance patient safety.

## 1. Introduction

Recent advances in pharmacology have introduced new drugs to treat various conditions, significantly increasing the concomitant use of multiple drugs. This trend increases the risk of drug-drug interactions (DDIs) that represent an increasing challenge in modern healthcare, particularly as the prevalence of polypharmacy rises among aging populations and patients with multiple comorbidities [1]. While dentists typically use a limited range of pharmacotherapeutic classes [2,3], the potential for DDIs remains a significant concern, particularly in patients receiving treatment for acute or chronic comorbidities [4]. These drug interactions can compromise treatment effectiveness, increase the risk of complications, or aggravate adverse drug reactions, which may impact both dental procedures and systemic health.

Several pharmacological agents are routinely employed in dental care for local anesthesia, infection control, pain management, and procedural sedation. Local anesthetics, such as lidocaine, mepivacaine, and articaine, are widely used in dental procedures via topical or injectable formulations [3,5,6]. They are often combined with vasoconstrictors like epinephrine to prolong anesthesia and reduce bleeding through local vasoconstriction [5]. Despite ongoing debate, antibiotics, including amoxicillin and clindamycin, are occasionally used prophylactically in dental surgery [7]. While amoxicillin may reduce implant failure risk [8], clindamycin and metronidazole are also effective for periodontal and postoperative infection control but require cautious use due to side effects and allergy risks [9,10]. NSAIDs (or acetaminophen for NSAID-intolerant patients) are the first-line agents for postoperative dental pain [11,12]. Corticosteroids and NSAIDs help reduce surgical inflammation [13,14], while opioids such as codeine and tramadol, often combined with acetaminophen, are reserved for more severe pain [11,15]. Sedation is effective in reducing dental anxiety and enhances patient compliance during procedures [16]. Benzodiazepines are effective oral anxiolytics [17,18], while nitrous oxide and intravenous midazolam or propofol are used for moderate to deep sedation in complex cases [19,20,21].

To reduce DDI-related risks, dentists should communicate clearly with patients about all current medications, including over-the-counter and herbal products. When managing medically complex cases, drug interaction checkers and consultation with prescribing physicians are also recommended. Despite the clinical importance of DDIs in dentistry, published data on their prevalence and impact remain scarce. The absence of standardized guidelines for identifying and managing these interactions may contribute to inconsistent prescribing and increase the risk of adverse outcomes. Given the growing complexity of pharmacotherapy, further investigation into DDIs in dental practice is essential to prevent iatrogenic harm and improve patient safety.

Most existing studies focus on DDIs in general medical settings [1,4,22] or report adverse reactions to drugs commonly used in dentistry [3]. Some explore interactions between dental drugs and commonly dispensed medications [23] or with herbs, foods, and other substances [24]. Others examine clinically relevant DDIs involving hepatic cytochrome P450 pathways or focus on specific drug classes such as antithrombotics [25], oral anticoagulants [26], disease-modifying antirheumatic drugs [27], and antihypertensives [28]. More recently, the use of remdesivir in COVID-19 patients has prompted analysis of its potential interactions with dental drugs [29].

Few studies have used real-world data (RWD) from clinical dental settings, where medication profiles reflect the complexities of everyday practice, thus limiting our understanding of DDIs’ impact on dental treatment, particularly in patients with polymedication or chronic conditions. While Mohan et al. [30] provided an overview of potential DDIs relevant to dental practice, their work mainly synthesized theoretical knowledge without providing real-world prevalence data. Goh et al. [31] focused on developing digital decision-support systems for DDI detection in dental clinics but did not analyze patient-level clinical data. A retrospective study of emergency and dental clinic records found that over 50% of patients had systemic diseases, with analgesics being the most frequent cause of DDIs [32]. Interest in DDIs among elderly patients is reflected in the article [33], which reports nearly 10% major DDIs in patients aged 60 and older using the drugs.com; however, their analysis did not address the underreporting. Similarly, a pilot study in 100 dental patients found high rates of medication discrepancies in those over 65, highlighting the risk of mismanagement due to incomplete drug histories [34]. However, neither study assessed the severity or clinical impact of the identified interactions.

To address the literature gap in drug-drug interactions (DDIs) in stomatology, our study has the following specific objectives: (i) to quantify the prevalence of DDIs in dental patients with comorbidities; (ii) to assess the severity of DDIs, focusing on interventional, post-interventional, and ongoing drugs for comorbidities; (iii) to evaluate the clinical relevance of these DDIs, with an emphasis on their impact on patients with cardiovascular comorbidities, and (iv) to develop evidence-based recommendations for safe analgesia and anesthesia in dental practice, informed by the prevalence, severity, and clinical relevance of DDIs. We systematically assessed potential interactions between dentistry-specific drugs and those for acute or chronic conditions using the DrugBank Drug Interaction Checker online tool [35]. To the best of our knowledge, this is the first study conducted in a cohort of Romanian dental patients, providing valuable insights into the prevalence and nature of DDIs at the intersection of dental and systemic pharmacotherapy and clinical implications in dental care.

## 2. Materials and Methods

### 2.1. Study Design and Ethical Approval

We performed a retrospective study on patients of a private dental practice in Timișoara from November to December 2024. The “Victor Babeș” University of Medicine and Pharmacy Timișoara Scientific Research Ethics Committee approved this study protocol (no. 60/2022).

To evaluate DDIs, we included only patients with medical records documenting at least two drugs. Therefore, inclusion criteria were (i1) patients providing written informed consent for data use (or parental consent for those <18 years), (i2) patients of any age (≥18 years, or <18 with parental consent), (i3) undergoing dental procedures, and (i4) medical records documenting at least two drugs, including interventional, post-interventional, or ongoing drug therapies, to enable DDI analysis. Exclusion criteria were (e1) patients without informed consent (or parental consent for minors), (e2) patients under 18 without parental consent, (e3) patients with incomplete medication records, and (e4) those not receiving dental procedures during the study period. We enrolled 105 consecutive patients meeting these criteria to minimize selection bias. We systematically collected age, gender, dental diagnoses, procedures, interventional/post-interventional drugs, and chronic/acute conditions with corresponding therapy data from the patient dental file and patient self-reported forms (regarding comorbidities and additional drugs). We strictly ensured patient data confidentiality throughout the study.

### 2.2. Testing the Severity Level of Drug-Drug Interactions

We used the DrugBank Drug Interaction Checker tool to test the severity level of each possible drug pair for each patient. The DrugBank Drug Interaction Checker was used to test the severity level of each possible drug pair for each patient. We selected DrugBank to analyze DDIs, considering the following key strengths: (i) it is a comprehensive database that includes over 1.3 million DDI pairs, widely used in pharmacological research [6]; (ii) it is a free academic drug database, unlike other subscription-based tools; (iii) it provides a freely available API that reduces manual errors, facilitating rapid identification of interactions, particularly valuable for large datasets in research settings [36]; (iv) DrugBank is a versioned database that enables the analysis of the evolution of knowledge over time [37].When testing a drug pair, the DrugBank checker categorizes the drug-drug interaction severity as major, moderate, or minor, providing a brief description and bibliographic references. However, when DrugBank returns “No interactions found” for a drug pair, the tool cautions that this does not definitively indicate the absence of interaction between the two drugs.

### 2.3. Statistical Analysis

Descriptive statistics were applied to examine the dental patient cohort, summarizing demographic characteristics, medical history, dental procedures, prescribed medications, the prevalence and severity of drug-drug interactions, and their distribution among patients.

Statistical analyses were conducted to investigate the relationship between patient age, comorbidity status, and the prevalence and burden of DDIs. A Chi-square test of independence assessed whether the prevalence of major DDIs differed significantly across age groups (0–30, 31–60, and ≥61 years (statistical significance set at *p* < 0.05). The Kruskal-Wallis test compared the distribution of total DDIs across age groups and the Mann-Whitney U test compared total DDIs between patients with and without CVD (statistical significance set at *p* < 0.05).

This study included all consecutive eligible patients (*n* = 105) treated for two months at a single dental clinic. We designed this study as an exploratory, real-world analysis and did not perform a preliminary sample size calculation. However, we performed a post-hoc power analysis to assess statistical power retrospectively, which we will detail in Section 4. Sample size calculations for future studies aimed to achieve 80% power at a significance level of α = 0.05, assuming equal group sizes and using observed effect sizes as a reference.We use these results to determine the minimum sample size needed for future studies.

Statistical analyses were carried out using Python 3.10, with SciPy and pandas libraries for data processing and inferential testing.

Cross-validation with additional DDI checkers or literature sources was not performed, a limitation argued in the Section 4.

## 3. Results

### 3.1. Patient Demographics and Preexisting Clinical Profile

In this study, we compiled a dental patient dataset to examine demographic characteristics and preexisting diseases. The dataset includes patients aged 6 to 78 years, with a mean of 43.2 years and a standard deviation of 15.9 years. Table 1 shows the three age groups—young, middle-aged, and elderly—to deliver a broad overview of the cohort’s age distribution and the prevalence of DDIs. Furthermore, the dataset reveals that 48 patients (45.7%) reported preexistent chronic or acute diseases during their dental visits, with a summary of these disease categories reflecting the diverse health challenges within this population.

### 3.2. Dental Patient Profile

Our study provides an overview of the dental-related patient profile within our cohort, as presented in Table 2. The first two columns offer a picture of the prevalence of various dental diagnoses. The third and fourth columns present the dental procedures and their prevalence, highlighting the variety of dental care needs and procedures applied to our patient cohort.

### 3.3. Drug-Drug Interaction Analysis

We evaluated 1332 drug pairs using the DrugBank Drug Interaction Checker and identified 542 drug-drug interactions (DDIs) within our dental patient cohort. According to DrugBank, the remaining drug combinations showed no interactions. Table 3 shows that 25.0% of DDIs are moderate, indicating drug pairs requiring cautious co-administration, potential dose adjustments, or monitoring, though not necessarily discontinuation. Minor DDIs are less clinically significant and typically require minimal intervention, accounting for 13.4% of the tested drug pairs. We identified 2.3% of DDIs as major, according to DrugBank. Although major DDIs are less common, they may exhibit significant risks to patient health, and their severity needs thorough assessments, particularly for patients with complex medical profiles.

**Figure 1:**
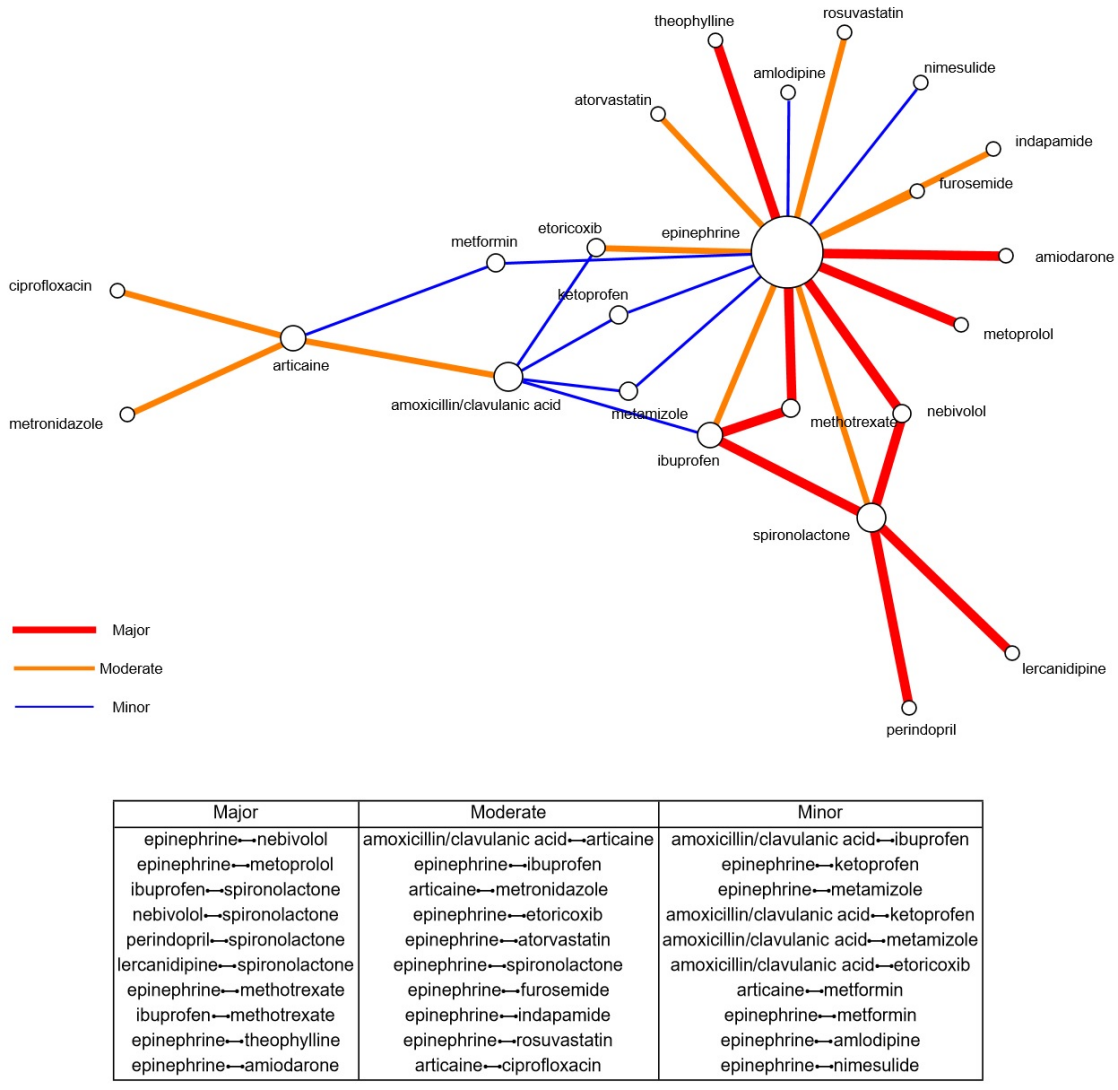
Top 10 drug-drug interactions. The upper panel illustrates the drug-drug interaction network built in Mathematica 13.0 from the top 10 drug-drug interactions (DDIs) at each severity level. Each node represents a drug, labeled by name, with its size proportional to its degree (i.e., the number of interactions with other drugs). Links between nodes indicate interaction relationships, with color and thickness denoting severity: thick red lines represent major DDIs, medium-thick orange lines indicate moderate DDIs, and thin blue lines correspond to minor DDIs. The lower panel illustrates the top 10 drug-drug interactions ranked by prevalence in our dental patient dataset, categorized as major, moderate, and minor DDIs.

Figure 1 illustrates the top 10 drug-drug interactions (DDIs) identified within our dental patient dataset, extracted from an analysis of 1332 drug pairs tested with the DrugBank Drug Interaction Checker. We ranked the major, moderate, and minor DDIs based on their prevalence in our cohort. To further support the interpretation of these findings, Figure 1 presents a visual drug-drug interaction network, built using Mathematica 13.0, of the top 10 DDIs at each severity level. Each node represents a drug labeled with its name; its size is proportional to its degree (i.e., the number of interactions with other drugs). The links between nodes indicate interaction relationships, with color and line thickness denoting the interaction severity: thick red lines represent major DDIs, medium-thick orange lines indicate moderate DDIs, and thin blue lines correspond to minor DDIs. This network visualization provides an intuitive overview of the most frequently interacting drugs and their foremost roles in dental care’s DDI landscape. Our findings highlight the frequency and variety of DDIs in dental practice and offer critical insights into the most common DDIs in this medical field.

**Figure 2:**
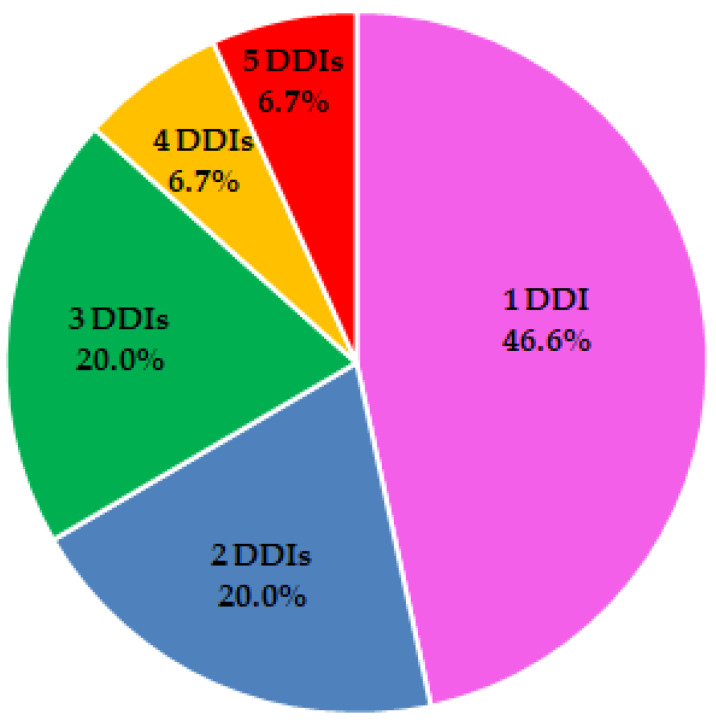
Distribution of major DDIs. The pie chart depicts the percentage of patients experiencing between 1 and 5 major DDIs.

Furthermore, Figure 2 illustrates that the 31 major DDIs identified affect 15 patients, with our analysis revealing that 46.6% of these patients experience one DDI, 20.0% have two DDIs, 20.0% have three DDIs, 6.7% have four DDIs, and 6.7% exhibit five major DDIs.

Table 4 presents the number of patients in each age category and the corresponding distribution of major, moderate, and minor DDIs, providing insight into how interaction severity varies by age.

The distribution of DDIs was also analyzed based on different age groups and the presence of cardiovascular comorbidities.

The Chi-square test performed to compare the proportions of patients with major DDIs across three age groups revealed no major DDIs in the 0–30 age group, 15.3% in the 31–60 age group, and 31.6% in the ≥61 age group, indicating a significant association between the age group and the prevalence of major DDIs, $\chi^{2}$(2, *n* = 105) = 9.19, *p* = 0.0101.

**Figure 3:**
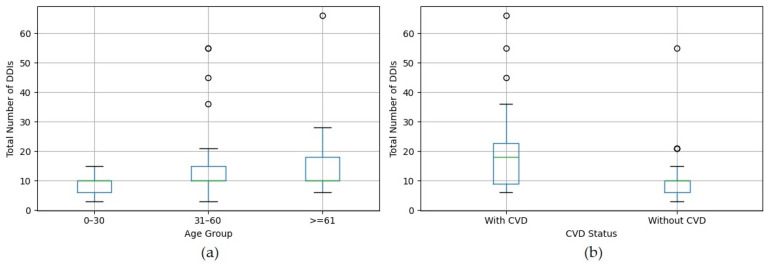
The distribution of total drug-drug interactions (DDIs) in your retrospective study. (**a**) Total DDIs by age group: patients aged 0–30 (*n* = 27, median 10, IQR 4), 31–60 (*n* = 59, median 10, IQR 5), and ≥61 (*n* = 19, median 10, IQR 8). (**b**) Total DDIs by cardiovascular disease (CVD) status: with CVD (*n* = 20, median 18, IQR 13.75) and patients without CVD (*n* = 85, median 10, IQR 4).

The Kruskal-Wallis test showed a significant difference in total DDI counts across age groups, with H(2) = 7.81, *p* = 0.0202; Figure 3a presents the boxplot of the total DDIs count by age group. While the median total DDIs counts remained consistent across age groups, the Kruskal–Wallis test revealed a significant difference in distributions, with older adults exhibiting broader interquartile ranges. Furthermore, the Mann–Whitney U test indicated a significant difference in total DDIs counts between patients with and without CVD (U = 490, *p* = 0.0033); Figure 3b presents a boxplot comparing the total DDIs count between patients with and without CVD, demonstrating a higher burden of DDIs in patients with CVD.

## 4. Discussion

Drug-drug interactions emerge from the simultaneous use of two or more drugs. DDIs may enhance therapeutic efficacy or lead to unexpected adverse reactions. They also may produce synergistic, antagonistic, or additive effects, affecting treatment effectiveness and patient safety. Increased life expectancy increases the prevalence of polypharmacy and consequently increases the exposure to DDIs [1,4,24,33,38]. In this context, physicians should exercise greater caution when prescribing treatments to minimize risks and provide a safe medical care [32,39].

Our analysis of preexistent diseases in the dental-patient dataset reveals a diverse range of comorbidities (see Table 1), with cardiovascular diseases (CVD) being the most prevalent at 19.0%. The high prevalence of CVD in our cohort aligns with findings from other studies on dental populations, highlighting a common comorbidity burden in this clinical setting [2,32,33]. Given that CVD frequently requires long-term pharmacotherapy, patients in this category are at higher risk for DDIs, particularly with local anesthetics containing epinephrine and commonly prescribed NSAIDs [40]. The following most prevalent disorders are respiratory (7.6%) and hematological disorders (6.7%), emphasizing the significant burden of systemic health issues among dental patients, who may experience DDIs with commonly prescribed dental medications such as analgesics or antibiotics. Psychiatric and neurological conditions, each affecting 5.71% of the cohort, alongside diabetes mellitus (4.76%), further highlight the complexity of managing pharmacotherapy in these patients, as these conditions often require medications (e.g., antidepressants, anticonvulsants, or antidiabetics) that could alter the dental treatment efficacy or safety [41]. Gastrointestinal (3.8%), urological (2.85%), and dermatological (1.9%) disorders are less frequent conditions that contribute to the overall multimorbidity profile. Systemic infections, otolaryngological issues, and allergies (each 0.95%, *n* = 1) indicate their potential to influence dental treatment outcomes. The distribution of morbidities emphasizes the need for an agreement on drug use in dental practice, using tools such as the DrugBank DDI checker to adjust therapies and mitigate risks, specifically in patients with complex systemic diseases.

As presented in Table 2, the dental diagnoses and procedures identified in our cohort provide a landscape of oral health challenges within this population and their potential implications for drug therapy management. Apical lesions (47.6%) are the most common diagnosis, which is consistent with recent meta-analysis reporting their global prevalence [42]. The following diagnoses are abscesses (18.1%) and pulpitis (15.2%), suggesting a dependence on pharmacotherapy—such as local anesthetics or postinterventional pain management—that could complicate polypharmacy scenarios. Tooth extraction is the most frequent procedure (53.3%), reflecting a high burden of severe dental pathology that requires surgical or restorative approaches. Tooth extraction and endodontic treatment (19.1%) often require analgesics, antibiotics, or NSAIDs, which may interact with drugs that patients take for their systemic comorbidities, thus increasing the risk of DDIs [43,44,45,46,47]. Dental implants (12.4%) further indicate a subset of patients undergoing complex procedures or managing chronic tooth loss, potentially requiring prolonged medication regimens that reqiure careful DDIs screening [48,49].

### 4.1. Clinical Management Strategies

The DDIs identified by the DrugBank DDI checker analysis in our dental patient dataset revealed several major interactions between medications commonly used in dental procedures and those prescribed for underlying systemic conditions. Epinephrine, frequently used in dental local anesthetics, appears in multiple high-risk pairs with beta-blockers (e.g., nebivolol, metoprolol, atenolol, bisoprolol, carvedilol); these DDIs present substantial risks for patients with cardiovascular comorbidities, potentially exacerbating hypertension—the local anesthetics containing epinephrine require cautious administration in patients on cardiovascular medications [5,22,23,40,50]. Epinephrine in dental anesthesia should be used at the lowest effective concentration, tailored to each patient’s clinical profile, as even low doses can influence cardiovascular function. Guimaraes et al. confirm that locally administered epinephrine can cause transient but clinically significant systemic effects, especially in sensitive individuals or those taking beta-blockers, even with proper technique, due to its vasoconstrictive properties [50]. Notably, around 20% of intraoral injections may transiently elevate systemic adrenaline levels, potentially contributing to complications such as ischemic events, arrhythmias, tremors, glycemic shifts, and exacerbation of drug interactions; these effects arise both from direct stimulation of adrenergic receptors and indirect disturbances in potassium balance [51]. Psychological stress from anticipating dental injections can also activate the sympathetic nervous system, mimicking epinephrine’s cardiovascular effects [51]. Therefore, some observed cardiovascular responses may reflect anxiety rather than solely drug interactions, further emphasizing the complexity of assessing epinephrine-DDIs in dental settings. In this context of potential epinephrine-associated DDIs, a practical and safe management strategy in dental settings is using mepivacaine, a well-tolerated and alternative local anesthetic [52]; although mepivacaine does possess mild vasoconstrictive properties, it may present a safer alternative in patients treated with beta-blockers, as it avoids the potent sympathomimetic effects of epinephrine [53]. For patients requiring prolonged anesthesia, phenylephrine may represent an alternative vasoconstrictor. As a selective alpha-1 agonist, phenylephrine avoids beta-adrenergic stimulation, reducing the risk of tachycardia and related cardiovascular effects. When used with lidocaine, it effectively prolongs the anesthetic duration and is appropriate for patients for whom epinephrine is not advisable [54]. Amiodarone and theophylline are cytochrome P-450 CYP3A4 substrates [6,55] with a narrow therapeutic index [6,56]; epinephrine, a CYP3A4 inhibitor, administered concomitantly decreases the metabolism of the CYP3A4 substrates, thus increasing the serum concentration, the therapeutic effect, and the cardiac adverse effects and toxicity [6]. Therefore, caution is advised when co-administering epinephrine with such drugs. A preoperative consultation is recommended for dental patients on CYP3A4-sensitive medications—particularly those with cardiovascular conditions. In these cases, vasoconstrictor-free anesthetics like plain mepivacaine offer a safer alternative [57]. Ibuprofen—a widely used analgesic in stomatology—can induce nephrotoxicity and consequently increase kalemia; when combined with spironolactone, it can exacerbate the hyperkalemia cardiovascular harmful effects [58,59]. Therefore, dentists should avoid or use ibuprofen with caution in patients receiving spironolactone and consider safer alternatives such as paracetamol/acetaminophen to minimize the severity of this DDI [6]. Ibuprofen-methotrexate combination leads to methotrexate accumulation and heightened toxicity of methotrexate because ibuprofen impairs its renal elimination [6]. In patients taking methotrexate, ibuprofen and other NSAIDs should be avoided due to the risk of impaired methotrexate elimination and toxicity. While acetaminophen carries a risk of major interaction, tramadol is a safer alternative, with only minor interaction potential [6]. The choice of analgesics should be individualized, and patients receiving high doses of methotrexate are advised to consult a rheumatologist or oncologist. Spironolactone is frequently prescribed with ACE inhibitors (e.g., perindopril and zofenopril in our dataset) and with angiotensin receptor blockers (e.g., candesartan cilexetil), combinations that increase the likelihood of hyperkalemia. In patients receiving spironolactone with ACE inhibitors or ARBs, dentists should avoid high doses of ibuprofen due to the increased risk of hyperkalemia and renal impairment and choose alternative analgesics such as paracetamol. If NSAIDs are required, the lowest effective dose for the shortest duration should be recommended, with close monitoring of renal function and serum potassium levels [40].

**Figure 4:**
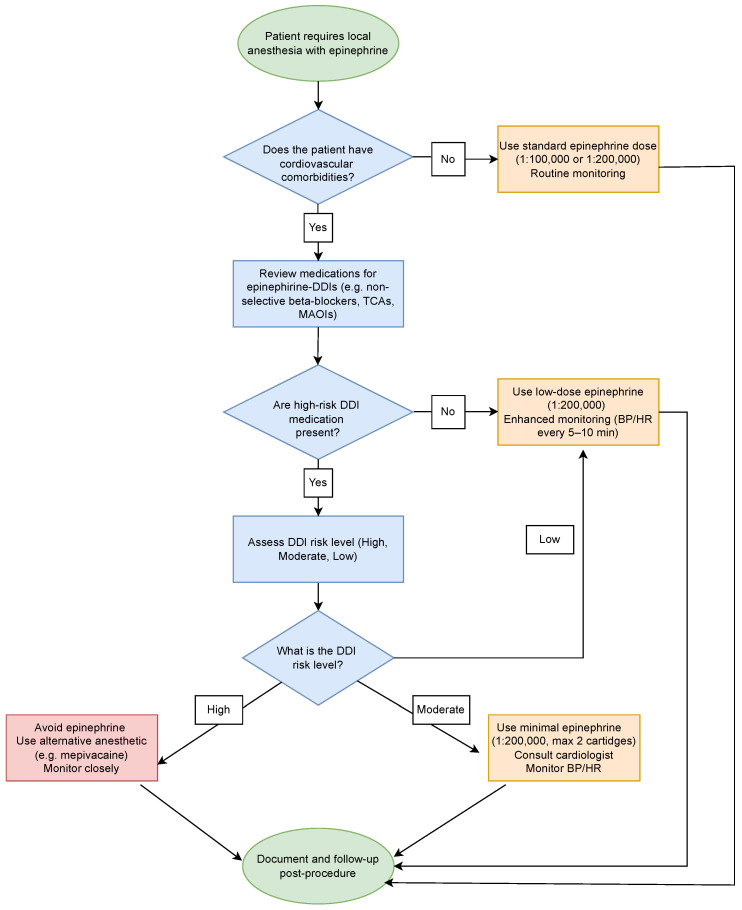
Decision flowchart for managing epinephrine use in dental patients with potential drug-drug interactions (DDIs). The algorithm assists clinicians in systematically evaluating cardiovascular comorbidities and medication profiles to determine the appropriate dosing strategy for epinephrine. Risk levels are categorized based on known DDIs with epinephrine. It provides personalized recommendations for using epinephrine or suggests complete avoidance in high-risk cases. The flowchart highlights the importance of enhanced monitoring, consultation when necessary, and documenting all decisions made after the procedure.

To manage epinephrine DDIs, we propose a decision flowchart presented in Figure 4. The algorithm guides clinicians through a stepwise evaluation of cardiovascular comorbidities and drug therapies to determine the epinephrine dosing strategy. Risk levels are stratified based on known epinephrine-DDIs—e.g., with beta-blockers, tricyclic antidepressants (TCAs), MAO inhibitors (MAOIs)—, with tailored recommendations for standard, low-dose/minimal epinephrine use or complete avoidance in high-risk cases. The flowchart emphasizes enhanced monitoring, consultation when needed, and documentation of all decisions post-procedure.

### 4.2. DDI Prevalence by Age and CVD

The pattern of major DDIs distribution presented in Figure 2 shows that almost half of patients encounter a single high-risk interaction; however, the other half face multiple major DDIs, amplifying the potential for adverse outcomes such as cardiovascular complications or heightened drug toxicity, particularly in a dental setting where drugs like epinephrine or ibuprofen are common. The occurrence of four to five major DDIs among 13.4% of patients highlights the complexity of managing polypharmacy in individuals with significant comorbidities. This finding is consistent with our cohort’s high prevalence of cardiovascular diseases (19.0%), which aligns with other studies involving dental patients, where beta-blockers and other high blood pressure-lowering drugs may produce DDIs with dental drugs. Therefore, DDI screening using tools like the DrugBank DDI checker is crucial for assisting clinicians in adjusting treatment plans and effectively reducing risks in routine dental care.

The major DDIs distribution across age groups in our dental cohort, as presented in Table 4, reveals the absence of major DDIs in the 0–30 age group in our cohort. This fact mirrors a good overall health status of the younger patient, who are less likely to have chronic diseases or polypharmacy, thus reducing their exposure to systemic medications that could interact with dental drugs. Dental treatments in this age range—often limited to caries management or minor procedures—may also involve fewer or less complex pharmacotherapies, minimizing the potential for severe DDIs. The 31–60 age group experiences the highest proportion of 61.3% of major DDIs. This result suggests that middle-aged patients combine active lifestyles with the onset of chronic diseases; as such, they may be exposed to a broader range of dental drugs while also starting antihypertensives or other systemic drugs for newly diagnosed conditions like hypertension, elevating the potential for DDIs. In contrast, the ≥61 years age group accounts for a lower proportion of 38.7% DDIs. While older patients are typically at higher risk for polypharmacy [60,61,62,63,64], the observed lower frequency of interactions than the 31–60 age group may indicate causes such as more cautious prescribing practices, better medication reviews [62,65,66], or differences in drug metabolism and clearance [67,68,69,70]. The observed lower prevalence of major DDIs in older adults (≥61 years) compared to the middle-aged group (31–60 years) in our study may be due to inconsistencies in medical records, especially with the underreporting of medications in older adults, which is consistent with findings from previous studies. For example, Abeleira-Pazos et al. identified significant inconsistencies in the medical records of older adults, remarking that incomplete or inaccurate drug histories often result from challenges in patient recall, especially in those with cognitive decline or complex regimens [34]. Similarly, Drenth-van Maanen et al. demonstrated that structured history-taking revealed discrepancies in the medication records of almost all participants, potentially leading to the underestimation of DDIs; furthermore, discrepancies in nonprescription drugs caused almost half of the consequences, thus accentuating the importance of including nonprescription drugs in the medication history taking [71]. Bennie et al. reported that over half of older adults in four of six European countries were prescribed ≥5 medications within six months, highlighting the widespread prevalence of polypharmacy, which typically increases DDI risk; this study also noted the frequent use of potentially improper drugs, such as proton pump inhibitors and benzodiazepines, suggesting that incomplete documentation of such drugs may obscure DDI detection [72]. Additionally, conservative prescribing in dental settings, prioritizing medications with lower interaction potential due to awareness of frailty, may further reduce observed DDIs. While the 31–60 group accounted for the largest share of all major DDI cases (61.3%), this reflects the distribution among affected patients. In contrast, when considering the entire cohort, the prevalence of major DDIs was actually higher in the ≥61 age group (31.6%) compared to the 31–60 group (15.3%), as shown by the chi-square test ($\chi^{2}$(2, *n* = 105) = 9.19, *p* = 0.0101). Figure 3 illustrates the pattern in the distribution of total DDIs across age groups and CVD status. Figure 3a shows that the ≥61 age group has a lower median total number of DDIs than younger age groups (0–30 and 31–60 years), supporting the hypothesis of underreporting or conservative prescribing in older adults. Conversely, Figure 3b illustrates a higher median total DDIs in CVD patients compared to those without CVD (18% vs. 10% in non-CVD patients). Still, the small sample size may limit the reliability of subgroup findings, particularly for the ≥61 age group. Therefore, the low number of major DDIs in this population should be interpreted cautiously, as it may not reflect a valid clinical trend but rather a limitation of statistical power. The chi-square test shows that the observed age-specific increase in the incidence of major DDIs highlights the importance of systematic medication review in elderly dental patients. Although no major DDIs were found in patients aged 0–30 years, a marked increase was observed in 31–60 and ≥61 age groups, reflecting the greater complexity of pharmacological regimens with advancing age; these findings highlight that elderly patients, despite sometimes underreporting their drugs, remain particularly vulnerable to clinically relevant DDIs. Therefore, incorporating routine DDIs screening tools and closer collaboration with physicians and pharmacists is crucial in managing middle-aged and older patients undergoing dental procedures. However, generalization of these results requires further validation in larger, more diverse, age-stratified cohorts.

A post-hoc power analysis of the Chi-square test for age groups in the current study indicated an achieved power of approximately 78% (Cramér’s *V* ≈ 0.296), suggesting that the study was sufficiently powered to detect age-related differences in prevalence of major DDIs. In contrast, the post-hoc analysis of the two-group comparison (CVD vs. non-CVD patients) revealed lower statistical power (≈40%) despite a moderate effect size (Cohen’s *h* = 0.39), indicating that the study was underpowered to detect differences in this subgroup comparison robustly. Accordingly, preliminary sample size calculations suggest that future studies require approximately 100 patients per group (assuming equal group sizes) to achieve 80% power. These studies should also aim to develop predictive models for identifying high-risk patients and to improve clinical decision support systems in dental care.

### 4.3. Limitations

Our study has several limitations: (i) the cohort size (*n* = 105), determined by the number of consecutive eligible patients over two months, is too small for robust statistical analysis and does not allow for generalization of the findings [73]; we did not perform a preliminary sample size calculation, as the study was designed as an exploratory, real-world analysis; (ii) the accuracy of patient self-reporting can be questionable because some patients may fill out forms hastily, have incomplete knowledge of their medications, or intentionally skip reporting sensitive diseases (e.g., psychiatric disorders or tuberculosis) [74]; (iii) our study reports that DDIs rely solely on the DrugBank DDI checker; cross-checking with similar DDIs checking tools would allow clinicians to better assess their agreement and ensure better drug combination management for patient safety [36].

## 5. Conclusions

Our study on 105 dental patients reveals a substantial DDIs burden, with 542 interactions identified across 1332 drug pairs. Among these, 31 major DDIs affected 15 patients, with a significant age-specific distribution: none in the 0–30 group, most frequently in the 31–60 age group, and fewer in the ≥61 group.Statistical analysis showed significant variation in total DDIs across age groups, with older patients displaying a wider interquartile range. Patients with CVD had significantly more DDIs than those without, indicating greater pharmacological risk. Specific DDIs, such as epinephrine with beta-blockers, highlight risks during frequent procedures like tooth extractions. Using real-world data (RWD) and the DrugBank DDI checker, our study addresses a critical gap in the sparse literature on dental DDIs, demonstrating their prevalence and age-specific risks. These findings endorse systematic DDI screening, particularly for patients (≥61) due to their higher major DDI burden, and tailored pharmacotherapy for older patients (≥61), such as low-dose epinephrine to mitigate risks. The significant age-specific distribution of major DDIs remains a key finding, supporting targeted screening. Dentists should systematically check drug histories, use validated DDI tools, and collaborate with physicians and pharmacists to manage medically complex patients (45.7% with comorbidities).

As a natural continuance of this work, our future studies will aim to evaluate a larger, multicentric patient cohort to enhance statistical power, increase robustness and generalizability, validate age-specific trends, preliminary sample size calculation for hypothesis-driven analyses, and further investigate the association between cardiovascular disease and DDIs. Post-hoc analyses from our current study suggest that enrolling approximately 200 patients would enable detection of clinically meaningful differences in major DDI prevalence between CVD and non-CVD subgroups. Additionally, research should focus on developing predictive models for high-risk patients and enhancing clinical decision support systems in dental practice.

## Figures and Tables

**Table 1:** Dental patient dataset’s demographic characteristics and preexisting conditions. The first row categorizes patients into three age groups: young, middle-aged, and elderly. The second row details the gender distribution. The final row lists the disease categories reported by patients at the time of their dental visit. All data are expressed as numbers and percentages.

**Age (years), *n* (%)**	0–30	27 (25.7)
	31–60	59 (56.2)
	≥61	19 (18.1)
**Gender, *n* (%)**	Females	65 (61.9)
	Males	40 (30.1)
**Preexistent diseases, *n* (%)**	Cardiovascular	20 (19.0)
	Respiratory	8 (7.6)
	Hematological	7 (6.7)
	Psychiatric	6 (5.7)
	Neurological	6 (5.7)
	Diabetes mellitus	5 (4.8)
	Gastrointestinal	4 (3.8)
	Urological	3 (2.8)
	Dermatological	2 (1.9)
	Otolaryngological	1 (0.9)
	Systemic infections	1 (0.9)
	Allergic	1 (0.9)

**Table 2:** Dental-related patient profile. The first two columns detail dental diagnoses and the corresponding number and percentage of patients. The third and fourth columns present the dental procedures performed and the number of patients receiving them.

Dental Diagnosis	*n* (%)	Dental Procedure	*n* (%)
Apical lesion	50 (47.6)	Tooth extraction	56 (53.3)
Abscess	19 (18.1)	Endodontic treatment	20 (19.1)
Pulpitis	16 (15.2)	Dental implant	13 (12.4)
Edentulism	13 (12.4)	Endodontic retreatment	8 (7.6)
Dental caries	7 (6.7)	Caries treatment	4 (3.8)
		Surgical tooth extraction	2 (1.9)
		Endodontic microsurgery	2 (1.9)

**Table 3:** Severity level of drug-drug interaction according to DrugBank. The first column illustrates the severity scale of drug-drug interactions. The second column offers the distribution of drug-drug interactions in our cohort in numbers and percentages.

Severity Level	*n* (%)
Major	31 (2.3%)
Moderate	333 (25.0%)
Minor	178 (13.4%)
No interactions found	790 (59.3%)

**Table 4:** DDI distribution by age. The number and percentages of major, moderate, and minor drug-drug interactions (DDIs) corresponding to each age group.

Age Group	Major, *n* (%)	Moderate, *n* (%)	Minor, *n* (%)
0–30	0	54 (16.2)	31 (17.4)
31–60	19 (61.3)	197 (59.2)	115 (64.6)
≥61	12 (38.7)	82 (24.6)	32 (18.0)

## Data Availability

The data presented in this study are available upon request from the corresponding author due to the ethical restrictions.

## Abbreviations

The following abbreviations listed alphabetically are used in this manuscript:

ACE	Angiotensin converting enzyme
CVD	Cardiovascular diseases
DDIs	Drug-drug interactions
NSAIDs	Non-steroidal anti-inflammatories
RWD	Real-world data
